# Systemic Lupus International Collaborating Clinics Frailty Index predicts mortality in systemic lupus erythematosus patients: data from the Almenara Lupus Cohort

**DOI:** 10.1136/lupus-2025-001938

**Published:** 2026-06-12

**Authors:** Benny Harold Rashuamán-Conche, Rocio Violeta Gamboa-Cárdenas, Victor Román Pimentel-Quiroz, Anubhav Singh, Cristina Reátegui-Sokolova, Claudia Elera-Fitzcarrald, Samira Garcia-Hirsh, Cesar Pastor-Asurza, Zoila Rodríguez-Bellido, Risto Alfredo Perich-Campos, Graciela S Alarcón, Manuel Francisco Ugarte-Gil

**Affiliations:** 1 Rheumatology Department, Hospital Nacional Guillermo Almenara Irigoyen, EsSalud, Lima, Peru; 2 Grupo Peruano de Estudio de Enfermedades Autoinmunes Sistémicas, Universidad Científica del Sur, Lima, Peru; 3 Internal Medicine, Baptist Hospitals of Southeast Texas, Beaumont, Texas, USA; 4 School of Medicine, Universidad Nacional Mayor de San Marcos, Lima, Peru; 5 Marnix E. Heersink School of Medicine, The University of Alabama at Birmingham, Birmingham, Alabama, USA; 6 School of Medicine, Universidad Peruana Cayetano Heredia, Lima, Peru

**Keywords:** Lupus Erythematosus, Systemic, Mortality, Outcome Assessment, Health Care

## Abstract

**Objective:**

Frailty has been shown to predict damage accrual in patients with systemic lupus erythematosus (SLE), including those from Latin America. However, the impact of frailty on mortality has been scarcely evaluated, particularly in Latin American populations. The aim of this study was to evaluate frailty as a predictor of mortality in Latin American SLE patients.

**Methods:**

Patients from a single-centre 2012–2025 prevalent cohort were studied. Mortality was defined as the vital status according to the Peruvian Department of Health. Frailty was ascertained using the Systemic Lupus International Collaborating Clinics Frailty Index (SLICC-FI) as a continuous variable. Univariable and multivariable Cox regression models were done to estimate its impact on mortality. Multivariable models were adjusted for possible confounders. Sensitivity analyses were performed including data up to March 2020 to avoid the potential influence of the COVID-19 pandemic. Additionally, a Kaplan-Meier curve was calculated by frail status (frail, SLICC-FI >0.21; non-frail, SLICC-FI ≤0.21).

**Results:**

Four hundred and ninety-five patients were included, 456 (92.1%) were women and 96 (19.4%) were frail. Their mean (SD) age at diagnosis was 35.0 (14.0) years, their mean disease duration at baseline was 6.7 (6.1) years and their mean follow-up time was 8.5 (4.2) years. Fifty-six (11.3%) patients died during the follow-up. The SLICC-FI predicted mortality even after adjusting for possible confounders (hazard ratio (HR)=1.54 (CI 95% 1.11 to 2.14), p=0.010). This prediction capability remained on the sensitivity analyses (HR 1.85; (95% CI 1.30 to 2.61), p<0.001).

**Conclusion:**

Frailty, as ascertained with the SLICC-FI, was a predictor of mortality in a Latin American cohort of SLE patients. Frailty could be considered in the monitoring of SLE patients; possible interventions to modify it should be developed.

**WHAT IS ALREADY KNOWN ON THIS TOPIC**

- The Systemic Lupus International Collaborating Clinics-Frailty Index (SLICC-FI) has been found to predict hospitalisations, damage accrual and health-related quality of life in a primarily Mestizo lupus cohort: the Almenara lupus cohort. The SLICC-FI has been found to predict mortality in cohorts from other ethnic backgrounds.

**WHAT THIS STUDY ADDS**

- The SLICC-FI predicts mortality in a Latin American Mestizo lupus cohort even after adjusting for possible confounders.

**HOW THIS STUDY MIGHT AFFECT RESEARCH, PRACTICE OR POLICY**

- It underscores the importance of examining the role of frailty in clinical practice and assessing it as a potentially modifiable clinical feature that warrants targeted interventions.

## Introduction

Frailty is a state of decreased physiological multisystemic reserve that increases vulnerability to adverse health outcomes.^1^ It has traditionally been studied in geriatric populations due to its strong association with adverse health-related outcomes on them.^1^ However, independent of chronological age, frailty has also been identified in patients with chronic conditions such as HIV infection, depressive symptoms,^2^ osteoarthritis^1^ as well as in patients with systemic autoimmune diseases.^1^

Furthermore, in recent years, a new tool has been designed to assess frailty in patients with systemic lupus erythematosus (SLE),^1^ namely, the Systemic Lupus International Collaborating Clinics Frailty Index (SLICC-FI), which integrates variables used to measure disease activity, organ damage and quality of life in these patients.^3^ Hence, the SLICC-FI quantifies frailty based on cumulative health deficits.^3^ In the SLICC cohort where it was developed, an increase in the SLICC-FI score was associated with a higher risk of mortality.^3^ A similar finding has also been reported in the Dalhousie Lupus Clinic Registry (DLCR).^4^

However, the impact of frailty on mortality in Latin American SLE patients has remained underexplored, although frailty has been reported to predict several health-related adverse outcomes in these populations.^5^,^7^ Understanding the role of frailty in mortality risk could contribute to the development of targeted interventions to improve patient outcomes.^3^ The aim of the study was to evaluate frailty as a predictor of mortality in Latin American patients with SLE.

## Methods

The Almenara Lupus Cohort was initiated in 2012; the characteristics of this cohort have been previously reported.^5^ This cohort includes all patients enrolled since January 2012 while the cut-off point for data analysis was March 2025. This cohort has been approved by the Institutional Review Board of our institution (3474-OCID-G-RAA-ESSALUD-11, 271-CEI-CIDG-RAA-ESSALUD-13, 302-CEI-ICD-G-RAA-14, 3027-OCID-G-RAA-ESSALUD-15 and 4072-OCID-G-HNGAI-ESSALUD-2017). All participants gave written informed consent before enrolling into the cohort.

All patients enrolled in this cohort met the American College of Rheumatology (ACR) 1997 criteria^8^ or the SLICC 2012 criteria^9^ and were older than 18 years of age at cohort entry. Exclusion criteria were the presence of other systemic autoimmune disease, except for Sjögren’s disease and the antiphospholipid syndrome.

Mortality was defined as the vital status according to the information obtained from the Peruvian Department of Health. Frailty was measured as a continuous variable and as a dichotomous variable. Status of frailty was classified as frail (SLICC-FI >0.21) and non-frail (SLICC-FI ≤0.21).

The SLICC-FI was calculated at the baseline visit on 46 of the original 48 items. The item myocarditis/endocarditis was the only not included item because it had not been recorded in this cohort. Additionally, since mood and anxiety disorders were recorded as a single variable in our cohort, it was not possible to separate them into two distinct variables as recorded by the SLICC-FI. Therefore, both were included as a single item. As previously described, the SLICC-FI can be calculated when more than 80% of the health deficits are measured, a statement fulfilled in this study.^4^

Potential confounders were collected and included demographic features (age at diagnosis, gender, socioeconomic status (SES), years of education, ethnic group, tobacco use), disease-related outcomes (disease duration, disease activity, ascertained with Systemic Lupus Erythematosus Disease Activity Index 2000 (SLEDAI-2K), and damage, ascertained with the SLICC/ACR damage index (SDI)) and treatment regimen (prednisone dose, antimalarial and immunosuppressive medication).

### Statistical analysis

Categorical variables were reported as numbers and percentages; continuous variables were reported as mean and SD. All variables were measured at the baseline visit. Univariable and multivariable Cox regression models were performed to estimate the impact of frailty on mortality; the main model included the SLICC-FI as a continuous variable per 0.05 increase and the alternative model included frail status as a categorical variable. Multivariable models were adjusted for possible confounders (age at diagnosis, gender, SES, ethnic group, tobacco use, disease duration, the SLEDAI-2K, the SLICC/ACR damage index, the Charlson comorbidity index, prednisone daily dose, antimalarials and immunosuppressive drugs use). Collinearity between the SLICC-FI and clinical variables such as the SLEDAI-2K, the SLICC/ACR Damage Index and the Charlson comorbidity index was assessed using the variance inflation factor. No significant interactions among these variables were identified. Additionally, the Kaplan-Meier curve and log-rank test were calculated by frail status.

Sensitivity analyses restricted to the follow-up period from 2012 to March 2020 were also performed to examine the relationship between frailty and mortality while avoiding the potential bias of mortality related to the COVID-19 pandemic.

Statistical significance was set at p<0.05. All analyses were performed using the SPSS 28.0 statistical package (IBM, Chicago, IL).

## Results

Four hundred and ninety-five patients were included; 92.1% were women. Frail patients constituted 19.4% of the participants. Table 1 includes the sociodemographic and clinical data for these patients. The mean (SD) follow-up time was 8.5 (4.2) years, and the mean SLICC-FI was 0.17 (0.04); during the follow-up period, 56 (11.3%) patients died.

**Table 1:** Baseline characteristics of SLE patients (n=495)

Variable	Number (%) or mean (SD)
Gender, women	456 (92.1)
Age at diagnosis, years	35.0 (14.0)
Socioeconomic status	
Low-medium low	68 (13.7)
Medium	157 (31.7)
Medium high-high	269 (54.3)
Ethnic group	
Mestizo	488 (98.6)
African Latin American	4 (0.8)
White	3 (0.6)
Tobacco use	
Never	389 (78.6)
Past	84 (17.0)
Current	22 (4.4)
Disease duration, years	6.7 (6.0)
SLEDAI-2K	3.1 (3.8)
SDI	0.9 (1.3)
Prednisone daily dose, milligrams	6.5 (7.0)
Antimalarial use	
Never	36 (7.3)
Past	59 (11.9)
Current	400 (80.8)
Immunosuppressive drug use	
Never	147 (29.7)
Past	103 (20.8)
Current	245 (49.5)
Charlson Comorbidity Index	1.34 (1.64)
SLICC-FI	0.17 (0.04)
Frail patients	96 (19.4)
Follow-up time, years, mean (SD)	8.5 (4.2)
Mortality during follow-up	56 (11.3)

Immunosuppressive drugs: mycophenolate mofetil, tacrolimus, cyclosporine, cyclophosphamide, leflunomide, azathioprine and rituximab. Value ranges considered: SLEDAI-2K (0–105 points), SDI (0–46 points) and Charlson Comorbidity Index (0–31 points).

ACR, American College of Rheumatology; SDI, SLICC/ACR Damage Index; SLEDAI-2K, Systemic Lupus Erythematosus Disease Activity Index 2000; SLICC-FI, Systemic Lupus International Collaborating Clinics-Frailty Index.

**Figure 1:**
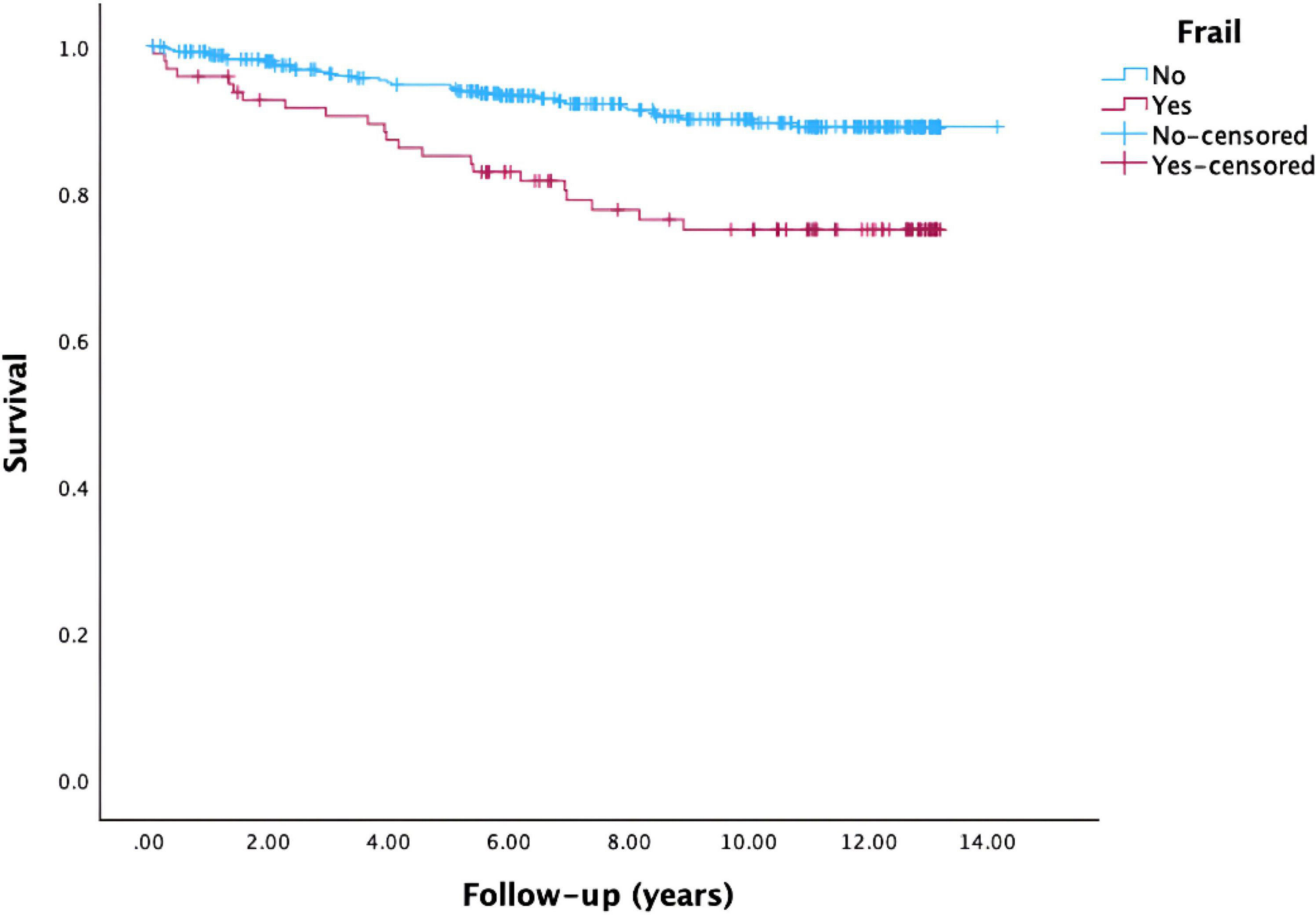
Survival status by frail status in patients with systemic lupus erythematosus.

Higher SLICC-FI scores at baseline predicted a higher mortality in the adjusted analysis (hazard ratio (HR)=1.54 (95% CI 1.11 to 2.14), p=0.010). These data are presented in table 2. The Kaplan-Meier curves are shown in figure 1 demonstrating the association of frailty with mortality (log-rank; p<0.001). However, frail status (frailty approached as a categorical variable) did not remain associated with mortality after adjusting for confounders (HR 1.41 (95% CI 0.75–2.64), p=0.279).

**Table 2:** Prediction capability between the SLICC-FI and the risk of mortality in patients with systemic lupus erythematosus. Unadjusted and adjusted models*

	Unadjusted			Adjusted		
	HR	(95% CI)	P value	HR	(95% CI)	P value
Main Model						
Baseline SLICC-FI (per 0.05 increase)	1.89	(1.47 to 2.43)	<0.001	1.54	(1.11 to 2.14)	0.010
Sensitivity analyses						
Baseline SLICC-FI (per 0.05 increase)	1.59	(1.18 to 2.14)	0.002	1.85	(1.30 to 2.61)	<0.001

* Cox regression multivariable models were adjusted for age at diagnosis, gender, SES, ethnic group, tobacco use, disease duration, SLEDAI-2K, SLICC/ACR damage index, prednisone daily dose, antimalarials and immunosuppressive drugs use.

ACR, American College of Rheumatology; HR, hazard ratio; SES, socioeconomic status; SLEDAI-2K, Systemic Lupus Erythematosus Disease Activity Index 2000; SLICC-FI, Systemic Lupus International Collaborating Clinics-Frailty Index.

In the sensitivity analyses, 296 patients were included. The follow-up period was cut to March 2020; mortality was 14.5% in these patients. The SLICC-FI prediction capability remained in the adjusted analyses (HR 1.85; 95% CI 1.30 to 2.61). These data are depicted in table 2.

## Discussion

Frailty at baseline predicted mortality in the Almenara Lupus Cohort. This effect remained even after multivariable adjustment and sensitivity analysis. Additionally, to our knowledge, the present study is the first one to evaluate the impact of the SLICC-FI on mortality within a primarily Mestizo lupus cohort.

The effect size between the SLICC-FI scores and mortality risk we found is comparable to the ones reported in the original SLICC inception cohort (HR=1.59, 95% CI 1.35 to 1.87, p<0.001) and in the DLCR (HR=1.31, 95% CI 1.01 to 1.70, p=0.04), both derived from multivariable-adjusted analyses.^3 4^ This relationship is also consistent with findings in other systemic autoimmune diseases. Regarding rheumatoid arthritis, both the Scottish Early Rheumatoid Arthritis (SERA) frailty index and the frailty phenotype model predicted mortality even after adjustment for confounders (HR 4.14, 95% CI 1.49 to 11.51 and HR 2.30, 95% CI 1.71 to 3.10, respectively).^10^ Regarding antineutrophil cytoplasm antibody-associated vasculitis, frailty ascertained with Rockwood Clinical Frailty Scale predicted mortality after adjusting for confounders (HR 1.90 (95% CI 1.03 to 3.52)).^11^

While the ability of SLICC-FI to predict damage accrual in SLE is well established,^3^,^5^ the index’s predictive value for mortality in our cohort appears to extend beyond the SDI per se.^3^ Several pathophysiological mechanisms could explain this effect in this group of patients. Principally, cellular senescence via immuno-mediated inflammation, sarcopenia and pharmacological effects via glucocorticoids or polypharmacy effect are the main proposed putative mechanisms.^1 12^

Additionally, frailty predicted admission to the emergency department^13^ and hospitalisations in our cohort.^7^ Furthermore, in a recent systematic review, which included 17 studies, frailty was found to be associated with damage accrual, hospitalisation and healthcare utilisation, mortality (from two studies of primarily non-Mestizo populations), lower health-related quality of life (HRQoL), higher disability and fatigue, vertebral fractures and subclinical cardiovascular disease. Most of these studies used the SLICC-FI, but others used the Fried phenotype and FRAIL scale.^14^ Therefore, this theoretical framework could effectively explain these attributes of the SLICC-FI on health-related outcomes.

Our study has some limitations; first, the research was conducted in a single tertiary centre; however, the Almenara Lupus Cohort is one of the largest Latin American SLE cohorts.^5^ Second, frailty was assessed only at baseline; therefore, the longitudinal changes in its prediction capability could not be explored. Nevertheless, this is an important first step to investigate the role of frailty in a Latin American SLE cohort, which has specific genetic, socioeconomic and clinical phenotypic characteristics.^5^ Third, this study used only one method to assess frailty in SLE (deficit accumulation model); therefore, the results might be different if other approaches were used. Nevertheless, the deficit accumulation model has more relevance in SLE, reinforcing the importance of our study.^1^

Consequently, recognising frailty as a key determinant of mortality in patients with SLE underscores the urgent need for targeted clinical interventions. Routine assessment of frailty should be considered an integral component of disease monitoring in clinical practice.^1^

In conclusion, frailty, ascertained with the SLICC-FI, predicted mortality in patients from a Latin American SLE cohort. Its impact on SLE outcomes needs to be evaluated in studies from different settings. Clinical interventions to improve frailty should be developed because of its potentially modifiable nature.
